# The Absorption of Fees

**Published:** 1907-01

**Authors:** Geo. H. Wells

**Affiliations:** Augusta, Ga.


### THE ABSORPTION OF FEES.

BY DR. GEO. H. WELLS, AUGUSTA, GA.

(Georgia State Dental Society, 1906.)

Though I have given you several lectures upon Chemistry and Metallurgy, I find that I have again been appointed a member of that committee.

In fact, I have been a member of that committee nearly as many years as I have been a member of the Society, except one year when you tried me as your second vice-president.

Somehow, you were not well pleased with me in that position, as you seemed to be, to have me give you talks upon Chemistry and Metallurgy. So here I am back again on that committee.

I do not know what was the matter with me as second vice-president. I tried to look as pleasant as it was possible for me to do, but somehow I did not look pleasant enough. Then, I had a nice lot of stationery given me for my use in official matters of importance. (I am now using some of it for this paper.) As a matter of fact I had no use for it, in any official position. The only use I found for it was to write to all the folks at home. and all the people that I had ever heard of, to show them how I was growing. I liked the job.

I had nothing to do but look pleasant. I was not on any committee; had no papers to write; had no responsibility. Just could find fault and say what was going to happen *some* day.

I thought I was filling the office to perfection. However, there seems to be a difference of opinion upon that, and now I am getting back at you as a member of the Committee upon Chemistry and Metallurgy.

This may seem to be a long heading; I will try to have the tail shorter. The subject of my paper this year will be strictly upon chemistry. The title is, "The Absorption of Fees." A

purely chemical question. I will have nothing to say upon metallurgy, but will give that to you at the next year's convention. (Possibly there might be some metallurgy about fees, but I will not discuss that part this year, but save my ammunition for the future.)

I suppose all the other papers will be upon scientific questions, as they are generally called. I think this is a scientific question also. In fact, the greatest science of sciences.

It is a *science* to get large fees out of people that do not want to pay them, as well as from those that are willing to pay nearly what I think my services are worth to them, or to me. (I will not say which I value most.)

This is for the benefit of the patient, as well as for the upbuilding of the profession, just as much as the other sciences that will be given here.

Gentlemen, at these dental meetings we hear a great deal about our being the *cream* of the earth. After the first few meetings I attended I used to come home thinking it was so.

After my return and someone came in and wanted to know "What would I charge to fill this tooth?" or "What would I charge to pull out her teeth?" Possibly it was, "What would I charge to pull out her teeth and put in new ones? Did I charge for pulling when I made the plates?" Gentlemen! It is a great relief to be able to say—that I have very little of that now. These people that are "shopping" and want to get your prices for this, and that, you do not want at any price. I could not please them when I used to fool with them and I do not believe that any of you do. They will do you more harm than good. The best way to treat this kind is to be too busy to see them. (No matter if you have done nothing in a week.) Now, I mean this in sober earnest. My experience is that they want more than those who pay you double what these "shoppers" are willing to pay. They are unappreciative, and always comparing prices with others. If they find someone else that had a plate, or tooth filled, that was anything like what you had done for them, for less money they would hold it up against you, that you had robbed them; besides, they would conclude that it was not done well enough for the money you had charged and come back for some changes. Possibly the next year they would come back and tell you before an audience that "Your fillings were coming out," or "They could not wear the plate that you had made for them." I had one case where a girl came back to tell me that one of my fillings was out. I asked her how it happened. Said that "she was just blowing her nose and the filling dropped out." I do not remember what I did in that case. What would you do?

Now, you young fellows that have just gotten out of college or have been out for a year or so and in the estimation of some (I won't say who they are) think they know a sight more than the old fellows who have been out of college years and years. I came out of college myself once. I know what I am talking about. I remember the last lecture given to us by one of the professors. We were told that we knew so much more about dentistry than most of the men then in practice, that we would come into competition with. That we would find that they were weak in the various scientific questions relating to dentistry. But that we had better not say so. That it would be better to treat them in a deferential manner and listen to them. That when it came to conducting a practice they could give us some pointers. We must not hurt their feelings by showing them that we felt that we knew more than they did. Speaking for myself, I will say that I took that all in. I did not think that was "flattery." I thought it was just so. That same man is a professor now. I cannot say whether he has changed that lecture since or not. Have you not all heard about the same lecture? Did you not feel the same way? I am now one of the "old fellows." I do not want to tell you how much over twenty years it is since I started in to get rich from dentistry, and not work very hard either.

Now, what shall we do when one of these "shoppers" comes in? Go up in price. Double it. She may have the work done. If she does she will think it good work, and that would, and have taken pains to do the best you could. If she thinks she has paid a good price, she will brag about it, and that you are no cheap dentist but do good work. It is a fact that you can do twice as good work when you are not looking at the clock to see if you are spending more time on it than you can afford to for the money.

If you work under these last conditions it hurts your work, and you are working for a patient that knows that you have made too low a price and is watching to see that you do not slight the work. She is suspicious from the start.

Gentlemen, this question of fees is the most vital question that we can consider. It affects us personally most vitally. Can you think of any great scientific discovery by a member of the dental profession that would raise the general status of dentistry as much as to have a well paid set of professional men? Men that have means to become cultured, traveled men that could discuss all questions scientific, and subjects of general interest to the world at large.

It is next to impossible for a man to become cultured that has to work hard all day and every day in support of self and family. I repeat "That it is practically impossible for a man to work for small fees, and make any extensive researches in scientific questions relating to dentistry or anything else. His time and strength is taken up with the bare necessities of physical life. Granted that he has the ability, he has not the strength. There are plenty of men in dentistry that are stifled and smothered in the struggle for a respectable existence. Men that should amount to something. This is tragedy, not comedy. I know that we all can not become famous if we had the time and means that I could wish. But we can become men of intellectuality. Men that would be chosen to meet distinguished people visiting our towns and cities. How many times is a dentist chosen to meet and help entertain the city's visitors? We are far from reaching our full limit. It is with us to come nearer it. The first and necessary step is to have the means and time to become cultured men. The only way that this can be done is to put a value upon our skill and time that will accomplish it.

The world generally takes a man at the value he places upon himself. Think of it, and see if this is not so? I know that you will be able to think of men that occupy a greater position than you think your merits warrant, but you know that they have put a higher estimate upon themselves and it passes for coin.

It will benefit our patients also, as much as it does the profession. I state most positively "That it is impossible for a man to reach his highest ideals when he knows all the time that he is not going to be fully paid for his best efforts. That he is sacrificing his life and possibilities for culture in trying to do what is best for the patient all the time. That he is exhausting his strength so that he can do nothing for his own improvement.

Did a man ever accomplish anything who did not respect himself and put a high estimate upon his own ability and skill?

He must have self-confidence, and a great deal of it, to accomplish great things, and must expect a corresponding reward.

He does not retain his own self-respect when he charges a smaller fee than he knows the services are worth.

Do you not feel better and have a higher opinion of yourself when you have completed a good work and receive as good a fee for it?

Does it not inspire you to do better work next time?

It is a very material benefit to the patient to pay good prices for professional services rendered by the dentist.

They will get better services no matter who is doing the work.

To emphasize this I am ready to repeat it.

Now, it is not the people that are to blame because we do not get better renumeration; they formerly paid higher fees, and made no more complaint than they will if we continue downward in our scale of prices.

They are better able to pay them now and would be glad of better service than we can render, and will pay for it.

You can think of men that put a high value upon themselves and the people pay them without the complaints that they make against much smaller fees charged by others.

The best people don't buy the cheapest articles that they find in the stores. When they go in for the cheapest they are expecting "shoddy." Why should they think that dentistry is the only exception?

They don't do it. The people that want the best are expecting to pay for it. They don't buy the cheapest clothing.

The stores that are looking for the cheapest goods are not the stores that make the greatest successes.

They make "quality" the leading feature.

We should do the same. Prices should be a secondary consideration. Look around you. Do you not see that it is the men that are doing the best work that are getting the best prices as a rule?

Do you believe that they would be doing as good work if they were working for half their fees? I do not. We are human.

It is only possible for one to keep up to their highest ideal when they know that their efforts will be fully appreciated.

Some men seem to think that they will not get any work if they do not make as low (and generally lower) fees than those around them.

I know that I once told a fellow practitioner in the same city that I live in "That he was not getting good enough prices. That instead of going down in my price I was going up." His reply was, "That one would go to sleep and be left behind if he did that. That it could not be done." I have done it, I may be asleep, but the net results financially are better and I do more work.

I repeat to myself in this paper: Be true to ourselves. True to the upbuilding of the profession. To raise our profession to a higher standing. To be men of mark in our own communities. "Quality" must be our first aim.

